# Influence of Sepsis on Clinical Outcomes During Mechanical Circulatory Support by Microaxial Flow Pump in Patients with Cardiogenic Shock Following Acute Myocardial Infarction

**DOI:** 10.3390/jcm15103989

**Published:** 2026-05-21

**Authors:** Philip Düsing, Julia Markgraf, Baravan Al-Kassou, Marko Bulic, Thomas Beiert, Sebastian Zimmer, Nikos Werner, Felix Jansen, Georg Nickenig, Andreas Zietzer

**Affiliations:** 1 Heart Center, Department of Medicine II, University Hospital Bonn, 53127 Bonn, Germany; 2 Heart Center Trier, Medical Department III, Krankenhaus Der Barmherzigen Brüder, 54292 Trier, Germany; 3 Kardiologie Köln, Gemeinschaftspraxis Kardiologie, 50676 Cologne, Germany

**Keywords:** cardiogenic shock, sepsis, mechanical circulatory support

## Abstract

**Background:** Cardiogenic shock (CS) is characterized as a state of low cardiac output that is frequently associated with multisystem organ failure. For over two decades, revascularization of the culprit lesion remained the only interventional treatment option to improve outcomes in CS following acute myocardial infarction. However, recently published data provide evidence that the use of a microaxial flow pump for mechanical circulatory support (MCS) in STEMI-related cardiogenic shock significantly reduced mortality after 180 days. Increased rates of complications such as sepsis were observed under MCS. The present study aimed to investigate the influence of sepsis on prognoses in patients with CS receiving temporary MCS with a microaxial flow pump. **Methods and Results:** This retrospective cohort study included 38 patients who received a microaxial flow pump for CS between 2014 and 2017. All patients were analyzed for the presence of sepsis, defined as infection and an increase in the Sequential Organ Failure Assessment (SOFA) score of ≥2 points. Analyzed clinical outcomes included all-cause mortality after 30 and 365 days and changes in renal function. A total of 38 patients were included in the final analysis. The 30-day all-cause mortality was significantly higher in the sepsis group than in the no-sepsis group (53.9% vs. 8.3%, *p* = 0.014). The findings were consistent for mortality at 365 days (65.4% vs. 16.7%, *p* = 0.008). **Conclusions:** These results indicate that sepsis significantly increases the risk of all-cause mortality at 30 and 365 days among patients with CS following AMI and receiving MCS via a microaxial flow pump.

## 1. Background

Cardiogenic shock (CS) is defined as a condition of low cardiac output that results in end-organ hypoperfusion, which is frequently associated with multisystem organ failure [1]. Left ventricular dysfunction following acute myocardial infarction (AMI) represents the main pathology leading to CS and accounts for more than 80% of cases [2]. Other potential causes for CS are mechanical complications following AMI or non-infarct-related CS caused by decompensated chronic heart failure, arrhythmias, or other cardiac pathologies [2]. CS occurs in 5–10% of patients hospitalized following AMI, and the mortality remains high, with 40–50% within 30 days [2,3]. For over two decades, early revascularization of the culprit lesion remained the only treatment option to improve outcomes in CS following AMI [4]. However, the recently published results of the “DanGer Shock” trial provide evidence that the routine use of a microaxial flow pump for mechanical circulatory support (MCS), along with standard care, in STEMI-related cardiogenic shock significantly reduces all-cause mortality compared with standard care alone [5].

Temporary MCS can be achieved with a group of heterogeneous devices, and their use has increased in various clinical settings over recent years [6]. Percutaneous microaxial flow pumps (such as the Impella CP (Abiomed)) are placed via the femoral artery, drain blood from the left ventricle (LV) and deliver it into the ascending aorta with a flow of up to >3 L/min [5]. This leads to increased mean arterial pressure and, consequently, improved coronary and systemic perfusion [6,7]. In addition, the device “unloads” the left ventricle, reducing LV end-diastolic pressure and decreasing wall tension and myocardial oxygen demand [6]. The microaxial flow pump is used for the treatment of CS or to perform protected percutaneous coronary interventions (PCI) [8]. Despite reducing mortality in STEMI-related CS, the incidence of adverse events was increased with the use of the microaxial flow pump [5]. Adverse events observed under therapy with the microaxial flow pump included moderate or severe bleeding, limb ischemia, renal replacement therapy and sepsis with positive cultures [5,9].

According to the latest international consensus definition for sepsis (Sepsis-3), Sepsis is defined as a life-threatening organ dysfunction caused by a dysregulated host response to an infection [10]. Diagnostic tools for the detection of sepsis include the Sequential Organ Failure Assessment (SOFA). In patients with suspected infection, an acute change of ≥2 points in the total SOFA score indicates organ dysfunction and an overall mortality risk of approximately 10% [10]. In general, sepsis is associated with a mortality of 10–20% [11]. Interactions between sepsis and CS are complex and clinically significant. Sepsis is a frequently observed complication of CS reported in 15–20% of cases, and is associated with a higher mortality [12]. Due to vasodilatation, septic patients with CS are at high risk of developing mixed cardiogenic-vasodilatory shock [12]. Additionally, sepsis-induced left ventricular dysfunction, which results from inflammatory mediators, decreased responsiveness to adrenergic agents, and volume overload, may further complicate the treatment of patients with CS [13]. However, limited data exist investigating the influence of sepsis in a subpopulation of patients with CS under MCS. Sepsis occurs in over 30% of patients with CS treated with microaxial flow pumps [14,15]. While there are no randomized trials available investigating the use of the microaxial flow pump in septic patients, data signal a benefit of the use of VA-ECMO for patients with sepsis-induced cardiogenic shock [16]. The present study aimed to investigate the influence of sepsis on prognosis in patients with CS receiving MCS using a microaxial flow pump.

## 2. Methods

### 2.1. Study Design and Population

The present study is a retrospective cohort investigation. Patients who received an Impella CP for AMI-related CS at Heart Center Bonn between 2014 and 2017 were analyzed for the presence or absence of sepsis. The patients were analyzed from the time point of treatment in the cardiac catheterization laboratory, where percutaneous coronary intervention and implantation of the microaxial flow pump were performed according to institutional standards. Advanced CS was identified per SCAI criteria at presentation [17]. This includes patients with hypoperfusion that required pharmacological or mechanical support at presentation (SCAI C), patients with worsening shock despite escalation (SCAI D) and patients presenting with refractory shock or circulatory collapse (SCAI E). After the cardiac intervention, patients were treated in the cardiac intensive care unit in accordance with local standard operating procedures.

Patients were, therefore, divided into a “sepsis group” and a “no sepsis group”. Sepsis is characterized as a life-threatening organ dysfunction caused by an inadequate host response to an infection [10,18]. According to current guidelines, organ dysfunction is defined as a change in the SOFA score of ≥2 points [19]. Thus, patients in the sepsis group had to fulfil clinical criteria, such as a suspected infection, positive blood cultures or radiologic evidence of pulmonary infiltrates, in addition to a change in the SOFA score of ≥2 points within 24 h. Patients who died within 24 h or were treated for less than 24 h in the study center were excluded from the analyses.

### 2.2. Analyzed Outcomes

To determine the effect of sepsis on general outcomes, we screened for mortality and renal endpoints. Only patients with completely recorded data for 12 months after microaxial flow pump implantation were included. Renal endpoints included changes in estimated glomerular filtration rate (eGFR). For mortality analyses and clinical outcomes, we screened the medical records available at the study center. Laboratory investigations were obtained during routine clinical care, and these parameters were also obtained from clinical records. Hemolysis was identified by LDH ≥ 1000 U/L and Haptoglobin < 0.3 mg/dL in 2 consecutive blood samples within 24 h. Major bleeding complications were characterized by the GUSTO criteria as hemodynamic impairment with intervention or intracranial haemorrhage. Moderate bleeding complications were defined as requiring a blood transfusion but not resulting in a hemodynamic compromise [20]. Septic patients were further analyzed for maximum SOFA score, focus of infection, laboratory results, positive blood cultures and duration of antibiotic treatment.

### 2.3. Statistics

Statistical analyses were performed with the software Graphpad Prism (version 9) and IBM SPSS Statistics (version 29.0.2.0). Statistical details are displayed in the figure legends. Mortality and survival based on the presence of sepsis were estimated using the Kaplan–Meier method, and log-rank tests were used to determine statistical significance. Fisher’s exact test was used to assess differences in categorical variables, while a Student’s *t*-test or Mann–Whitney U test was applied for analyzing continuous variables depending on data distribution. Analysis of the time course of eGFR was performed by two-way ANOVA and Bonferroni’s multiple comparison test. Statistical significance was considered as a two-tailed probability value ≤0.05.

### 2.4. Ethics

This analysis was performed in accordance with the Declaration of Helsinki. Consultation with the local ethics committee of the University of Bonn revealed no ethical concerns.

## 3. Results

### 3.1. Patient Characteristics

A total of 38 patients were included in the final analysis (Table 1). 26 patients met the predefined sepsis criteria and were included in the sepsis group. 12 patients did not meet the criteria for sepsis despite some individuals displaying elevated inflammatory markers. The mean age was 68 ± 12.5 years in the overall cohort and was comparable in both groups (*p* = 0.97). The majority of patients (92.1%) were male. All patients were treated for CS following AMI and had a mean left ventricular (LV) ejection fraction of 26.7% ± 10.8. The microaxial flow pump was implanted before PCI in 71.1% of patients. Patients in the sepsis group showed a higher serum lactate (4.7 ± 3.8 vs. 2.5 ± 1.1 mmol/L, *p* = 0.01), indicating aggravated shock with impaired organ perfusion. Numerically, more patients in the sepsis group required cardiopulmonary resuscitation (CPR) (42.3% vs. 16.7%, *p* = 0.26) and intubation (42.3 vs. 25%, *p* = 0.48) before the procedure compared to the “no sepsis” group. In parallel, patients in the sepsis group were numerically more often classified as having CS in stadium SCAI C-E (57.6 vs. 25.0%, *p* = 0.09) [17]. These trends were not statistically significant. We observed a statistically significant difference in the rate of intubation 24 h after the procedure between the sepsis and no sepsis group (57.6% vs. 16.6%, *p* = 0.03). No patient was escalated on a concomitant therapy with VA-ECMO.

All patients in the sepsis group had some form of organ dysfunction according to the SOFA score. The highest SOFA score in this group was 14. Most of the patients required the use of vasopressors or positive inotropic support. Only 7 patients in this group had positive blood cultures during the observation period, and 85% of these patients had a pulmonary focus of sepsis. The average duration of antibiotic treatment was 9.8 ± 6.5 days (Table 2).

### 3.2. Mortality

**Figure 1:**
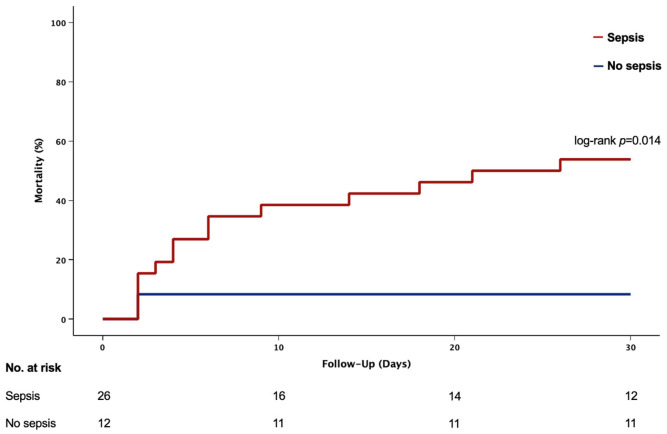
Death from any cause within 30 days in patients with CS treated with a microaxial flow pump and sepsis vs. patients without sepsis. Log-rank test.

The mortality from any cause after 30 days was 53.9% in the sepsis group compared to 8.3% in the no sepsis group (*p* = 0.014), as shown in Figure 1.

**Figure 2:**
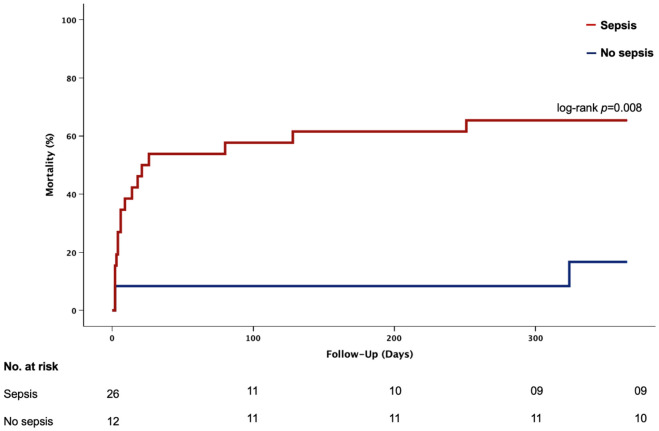
Death from any cause within 365 days in patients with CS treated with a microaxial flow pump and sepsis vs. patients without sepsis. Log-rank test.

Consistent results were observed for all-cause mortality after 365 days (65.4% vs. 16.7%, *p* = 0.008) (Figure 2).

### 3.3. Renal Outcomes

**Figure 3:**
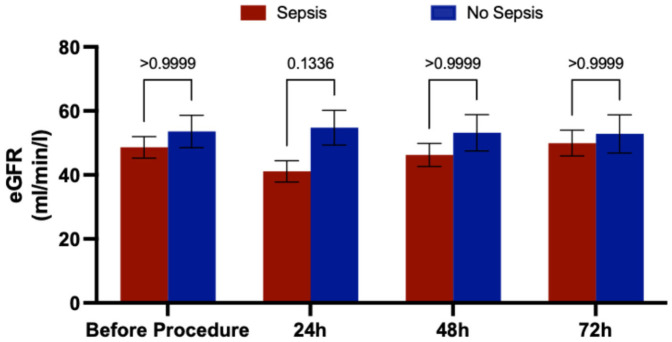
eGFR in septic patients and patients without sepsis. Two-way ANOVA with Bonferroni’s multiple comparison test for eGFR. eGFR, estimated glomerular filtration rate; RRT, renal replacement therapy.

We examined how sepsis affects renal function by investigating changes in estimated glomerular filtration rate (eGFR) within 72 h after implantation of the microaxial flow pump. We observed a trend toward decreased eGFR levels in the sepsis group at 24 h, which improved by 72 h (Figure 3). However, these changes did not show statistical significance.

### 3.4. Clinical Parameters and Complications During Hospitalization

Septic patients undergoing therapy with a microaxial flow pump for CS showed numerically prolonged duration of hospitalization (19.3 ± 13.2 vs. 11.3 ± 6.5 days), which was not statistically significant (*p* = 0.09), as presented in Table 3. The duration of microaxial flow pump support was short in both groups, with no significant difference observed between the sepsis and no sepsis group (1.8 ± 1.6 vs. 1.0 ± 1.3 days, *p* = 0.13). We further investigated the rate of major complications under therapy with the microaxial flow pump. No differences were observed in the incidence of hemorrhagic or ischemic strokes, bleeding events or ischemic peripheral events between the two groups. However, septic patients developed significantly more frequent hemolysis measured by increased LDH and decreased haptoglobin in 2 consecutive blood samples within 24 h (38% vs. 0%, *p* = 0.02).

## 4. Discussion

In this analysis, we investigated the influence of sepsis on outcomes in patients treated with a microaxial flow pump for CS following AMI. Our findings demonstrate that sepsis is associated with increased mortality in this high-risk population.

CS is a life-threatening state with a persistently high mortality rate between 40 and 50% within 30 days [2,3]. CS is defined as a state of low cardiac output, and the most frequent cause of CS is a decrease in myocardial contractility following AMI [1,2]. This may induce a pathophysiological spiral of decreased blood pressure, resulting in reduced coronary and tissue perfusion [1]. This aspect is well reflected by the study cohort, which had a mean left ventricular ejection fraction ≤30% following AMI. Emergency revascularization of the culprit lesion remains the major pillar in the treatment of AMI-related CS to improve outcomes [4]. However, in recent years, various MCS techniques have emerged to combat the pathophysiological spiral of CS. Among these, the use of a microaxial flow pump for LV unloading, in addition to standard care, has been shown to reduce mortality in STEMI-related CS [5]. In this study, however, the use of MCS was associated with an increased risk of adverse events, including sepsis [5].

In this retrospective cohort, we observed a statistically significant increase in absolute risk of 45.6 percentage points (*p* = 0.014) in the 30-day and 48.7 percentage points (*p* = 0.008) in 365-day all-cause mortality in patients with CS following AMI, treated with the microaxial flow pump, who developed sepsis, compared to those without sepsis. This finding is of relevance, as sepsis occurs in 10–40% of patients with CS treated with MCS [5,21]. Patients who developed sepsis required cardiopulmonary resuscitation numerically more frequently (42.3% vs. 16.7%), had more advanced CS based on SCAI classification and were more frequently intubated prior to MCS initiation (42.3% vs. 25%). As a result, patients in the sepsis group had a significantly higher serum lactate compared to patients in the no sepsis group (4.7 vs. 2.5, *p* = 0.01). Significantly more patients in the sepsis group were intubated 24 h after the intervention (57.6% vs. 16.6%, *p* = 0.03). In line with these observations, most patients in the sepsis group had a pulmonary focus (85%) of infection. Suspected or confirmed infection was recently identified as an independent risk factor for developing mixed shock (MS) in patients with CS [22]. MS occurs in every fourth patient presenting with CS and is associated with a higher hospital mortality [22]. In the “DanGer-Shock” trial, patients with CS treated with the microaxial flow pump were at higher risk of developing sepsis with positive cultures [5]. To date, there are no randomized trials available investigating the use of the microaxial flow pump in septic patients. A retrospective multicenter analysis showed improved survival in patients with sepsis-induced cardiogenic shock treated with VA-ECMO compared to controls not receiving ECMO [16]. Additionally, data from a meta-analysis suggest a potential benefit of VA-ECMO for patients with septic shock and low cardiac output refractory to inotropes [23]. Evidence for the concomitant use of a microaxial flow pump and VA-ECMO in septic patients is limited to case reports [24]. In the present cohort, no patient was escalated to concomitant treatment with VA-ECMO and a microaxial flow pump. This may be partly due to the fact that patients were enrolled between 2014 and 2017. The present study underscores the importance of prevention and screening for sepsis in patients with CS, which occurs in over 30% of patients [14,15]. A preventive antibiotic treatment with amoxicillin-clavulanate in patients after out-of-hospital cardiac arrest who were mechanically ventilated resulted in lower rates of ventilator-associated pneumonia while showing no difference regarding mortality [25]. The role of a preventive antibiotic regimen in patients with MCS needs to be addressed in future studies.

In the present investigation, we detected no significant differences in the duration of hospitalization (*p* = 0.09) or support with the microaxial flow pump (*p* = 0.11) between patients with and without sepsis. The most relevant complications during microaxial flow pump support include the risk of hemolysis, bleeding and peripheral ischemic complications [14,26,27]. In our cohort, we observed higher rates of hemolysis in septic patients undergoing MCS (*p* = 0.02). Hemolysis is a frequent finding among CS patients with MCS, which results from device- and patient-related factors [28]. The impact of hemolysis on outcomes such as mortality is currently unknown; however, the detection of hemolysis is important [29]. The present study is the first to demonstrate increased rates of hemolysis in septic patients receiving microaxial flow pump support, which may result from decreased left ventricular preload due to hypovolemia during sepsis [28].

Patients with CS who are treated with a microaxial flow pump are at high risk of developing acute kidney injury and requiring renal replacement therapy [30]. In our small cohort, we did not observe significantly impaired renal function in septic patients. However, future adequately powered studies may address the association of sepsis and acute kidney injury in patients with CS who are treated with a microaxial flow pump.

Our study has limitations. These include the observational design in a single-center setting with a relatively small sample size. Furthermore, the patient population was heterogeneous, with varying causes of AMI types and clinical scenarios. Residual confounding of length of hospitalization can not be ruled out. In addition, the current study only incompletely accounts for the time-dependent nature of sepsis. Due to these limitations, the present study cannot prove the causality of the exploratory findings.

## 5. Conclusions

In conclusion, sepsis was associated with an increased rate of 30-day and 365-day all-cause mortality in patients with CS following AMI treated with a microaxial flow pump as MCS. These data support that in clinical practice, close monitoring as well as early and consequent treatment of sepsis should be prioritized in patients with CS receiving MCS. This requires the identification of patients with CS and an increased risk of developing sepsis, such as requiring CPR or prolonged mechanical ventilation. Future studies should address whether enhanced surveillance strategies, including advanced hemodynamic monitoring, as well as optimized infection prevention and early antimicrobial treatment strategies, can improve survival in this complex patient population with CS under MCS and sepsis.

## Figures and Tables

**Table 1:** Characteristics of the study patients. Mean ± SD. BMI, body mass index; COPD, chronic obstructive pulmonary disease; PCI, percutaneous coronary intervention.

	Total (*n* = 38)	Sepsis (*n* = 26)	No Sepsis (*n* = 12)	*p*-Value
Median age—years	68.0 (±12.5)	67.8 (±16.0)	68.0 ± 10.5	0.97
Male sex—no. (%)	35 (92.1)	25 (96.2)	10 (83.3)	0.23
BMI	26.7 ± 3.2	27.1 ± 3.4	25.1 ± 0.7	0.22
Medical history				
Prior myocardial infarction—no (%)	9 (23.7)	4 (15.4)	5 (41.7)	0.11
Diabetes mellitus—no (%)	14 (36.8)	10 (38.5)	4 (33.3)	>0.99
Hypertension—no (%)	22 (57.9)	13 (50.0)	9 (75.0)	0.18
Hypercholesterolemia—no (%)	12 (31.6)	5 (19.2)	7 (58.3)	0.03
Chronic kidney disease—no (%)	11 (28.9)	9 (34.6)	2 (16.7)	0.44
Peripheral artery disease—no (%)	5 (13.2)	4 (15.4)	1 (8.3)	>0.99
COPD—no (%)	3 (7.9)	2 (7.7)	1 (8.3)	>0.99
Left ventricular ejection fraction—%	26.7 ± 10.8	25.6 ± 8.9	29.1 ± 13.6	0.45
Mean systolic blood pressure before procedure (mmHg)	118.1 ± 27.3	119.2 ± 29.8	115.9 ± 21.3	0.38
Mean heart rate before procedure (bpm)	91.9 ± 22.2	92.1 ± 26.9	91.6 ± 15.4	0.96
Mean lactate before procedure (mmol/L)	4.0 ± 3.3	4.7 ± 3.8	2.5 ± 1.1	0.01
Resuscitation before procedure—no (%)	12 (31.6)	11 (42.3)	2 (16.7)	0.26
Mean duration of Resuscitation—min	28.3 ± 21.2	26.4 ± 21.7	34 ± 18.7	0.55
SCAI stadium C-E—no (%)	18 (47.4)	15 (57.6)	3 (25)	0.09
Intubation before procedure—no (%)	14 (36.8)	11 (42.3)	3 (25)	0.48
Glascow Coma Scale at procedure	8.3 ± 5.6	7.2 ± 5.3	10.8 ± 5.3	0.07
Shock duration prior to procedure (hours)	2.8 ± 5.7	3.4 ± 6.7	1.6 ± 2.6	0.27
Intubated patients 24 h after procedure—no (%)	17 (44.7)	15 (57.6)	2 (16.6)	0.03
Acute myocardial infarction—no (%)	38 (100)	26 (100)	12 (100)	-
ST-elevation myocardial infarction—no (%)	14 (36.8)	12 (46.2)	2 (16.7)	0.15
Implantation of Impella before PCI—no (%)	27 (71.1)	18 (69.2)	9 (75.0)	0.69
Mean number of diseased coronary vessels—no	2.6 ± 0.6	2.7 ± 0.6	2.5 ± 0.6	0.45
Concomitant treatment with VA-ECMO—no (%)	0 (0)	0 (0)	0 (0)	-

**Table 2:** Characteristics of septic patients. Mean ± SD.

	Sepsis (*n* = 26)
Organ dysfunction—no (%)	26 (100)
Maximum SOFA-Score during sepsis	13.6 ± 4.5
Mean c-reactive-protein—mg/dL	149.1 ± 82.0
Mean leucocyte count—G/L	21.3 ± 6.3
Vasopressors/inotropic support—no (%)	22 (85)
Positive cultures—no (%)	7 (26.9)
Focus of infection	
Pulmonary—no (%)	22 (85)
Urinary—no (%)	3 (11.5)
Abdominal—no (%)	6 (23.1)
Unkown—no (%)	4 (15.4)
More than one focus—no (%)	12 (46.2)
Mean antibiotic treatment—days	9.8 ± 6.5

**Table 3:** Clinical parameters and complications of patients with and without sepsis treated with a microaxial flow pump for CS. Life-threatening or severe bleeding was defined as bleeding with hemodynamic impairment or intracranial haemorrhage. Moderate bleeding was defined as the need for transfusion of red blood cells without hemodynamic impairment. Hemolysis was identified by LDH ≥ 1000 and Haptoglobin < 0.3 in 2 consecutive blood samples within 24 h. CT, computed tomography. Fisher’s exact test for categorical variables, unpaired *t*-test for quantitative variables.

	Sepsis *(n* = 26)	No Sepsis (*n* = 12)	*p*-Value
Median duration of hospitalization *—days	19.3 (±13.2)	11.3 (±6.5)	0.09
Median duration of Impella support **—days	1.8 (±1.6)	1.0 (±1.3)	0.11
Complications			
Ischemic or hemorrhagic stroke confirmed by CT scan—no (%)	1 (4)	0 (0)	>0.99
Peripheral Ischemic complications requiring surgery or an intervention—no (%)	4 (15)	1 (8)	>0.99
Hemolysis—no (%)	10 (38)	0 (0)	0.02
Life-threatening or severe bleeding—no (%)	0 (0)	0 (0)	-
Moderate bleeding—no (%)	8 (31)	1 (8)	0.22

* In patients that survived >30 days. ** Patients with explanation post mortem excluded.

## Data Availability

The datasets analyzed for the current study are available from the corresponding author upon reasonable request.
